# Surface-modified elastomeric nanofluidic devices for single nanoparticle trapping

**DOI:** 10.1038/s41378-021-00273-y

**Published:** 2021-06-12

**Authors:** Deepika Sharma, Roderick Y. H. Lim, Thomas Pfohl, Yasin Ekinci

**Affiliations:** 1 Swiss Nanoscience Institute, 4056 Basel, Switzerland; 2 Biozentrum, University of Basel, 4056 Basel, Switzerland; 3 Laboratory for Micro and Nanotechnology, Paul Scherrer Institut, 5232 Villigen, Switzerland; 4 Institute of Physics, University of Freiburg, D-79104 Freiburg, Germany

**Keywords:** Nanofluidics, Nanoscience and technology

## Abstract

Our work focuses on the development of simpler and effective production of nanofluidic devices for high-throughput charged single nanoparticle trapping in an aqueous environment. Single nanoparticle confinement using electrostatic trapping has been an effective approach to study the fundamental properties of charged molecules under a controlled aqueous environment. Conventionally, geometry-induced electrostatic trapping devices are fabricated using SiOx-based substrates and comprise nanochannels imbedded with nanoindentations such as nanopockets, nanoslits and nanogrids. These geometry-induced electrostatic trapping devices can only trap negatively charged particles, and therefore, to trap positively charged particles, modification of the device surface is required. However, the surface modification process of a nanofluidic device is cumbersome and time consuming. Therefore, here, we present a novel approach for the development of surface-modified geometry-induced electrostatic trapping devices that reduces the surface modification time from nearly 5 days to just a few hours. We utilized polydimethylsiloxane for the development of a surface-modified geometry-induced electrostatic trapping device. To demonstrate the device efficiency and success of the surface modification procedure, a comparison study between a PDMS-based geometry-induced electrostatic trapping device and the surface-modified polydimethylsiloxane-based device was performed. The device surface was modified with two layers of polyelectrolytes (1: poly(ethyleneimine) and 2: poly(styrenesulfonate)), which led to an overall negatively charged surface. Our experiments revealed the presence of a homogeneous surface charge density inside the fluidic devices and equivalent trapping strengths for the surface-modified and native polydimethylsiloxane-based geometry-induced electrostatic trapping devices. This work paves the way towards broader use of geometry-induced electrostatic trapping devices in the fields of biosensing, disease diagnosis, molecular analysis, fluid quality control and pathogen detection.

## Introduction

High-throughput contact-free trapping of individual nano-objects in aqueous media has immense importance for dynamic, chemical, physical, and biological studies. Over the past decades, several techniques for single-particle studies have been introduced, allowing either active or passive confinement of single objects in a contact-free manner. Contact-free confinement of an object enables its trapping in an aqueous solution without its physical contact with adjacent surfaces of the device and thereby allows a better way of studying and understanding the dynamics of particles and their physical and chemical properties. Thus, it provides a robust means for investigating molecular activities, diagnostic efficiency, and material characteristics.

Conventional active particle confinement methods, such as optical tweezers^1–3^, magnetic tweezers^4–6^, and dielectrophoretic trapping^7–9^, provide direct control of the trapping strength and manipulation of the particle position. However, the particle trapping strength in the aforementioned methods is dependent on various properties of the particle, such as the refractive index, permeability and permittivity with respect to its surroundings, and is also proportional to the particle volume and field gradients. Thus, as the particle size decreases to the nanoscale, exceedingly large applied fields are required to confine it. Furthermore, the freedom of manipulating trap locations in active methods requires a complex setup and an external field source. Recently, some other active trapping methods were introduced to achieve particle confinement under ambient conditions for extended periods, such as anti-Brownian electrokinetic (ABEL) trapping^10–12^. To avoid system complexity and the presence of external field gradients, passive trapping methods such as hydrodynamic trapping, convex lens-induced confinement (CLIC)^13–15^ and geometry-induced electrostatic (GIE) trapping^16^ were introduced, which allow single particle confinement in an integrated micro/nanofluidic device based on the device geometry and the device surface and particle interactions.

Among various methods, GIE trapping has shown the potential to be a robust method for stable high-throughput contact-free confinement of nanoparticles down to 1 nm in diameter in an aqueous solution without the requirement of any external field^16–19^. GIE-trapping inside a nanofluidic device is achieved using the electrostatic interactions between charged device surfaces and like-charged nano-objects. Due to the repulsive forces between the like-charged device surface and the charged particle, a nanoparticle levitates inside the fluidic device at specifically tailored nanoscopic locations^20^. Tailoring the topography of one surface of a fluidic device with nanoindentations generates nanoscopic electrostatic potential traps between the two surfaces and allows nanoparticle trapping inside the nanoindentations^16,20^. For radial-symmetric electrostatic potential traps inside a fluidic device, one device surface is patterned with nanochannels imbedded with cylindrical nanopockets, which leads to the formation of potential wells inside the nanopockets^20^.

Conventionally, GIE-trapping devices are fabricated using glass or silicon substrates, which acquire a negative surface charge density in an aqueous environment (pH > 2.4) due to self-deprotonation of silanol (Si-OH) groups at the surface^21–24^. Since GIE-trapping devices can confine only like-charged nano-objects in a contact-free manner, SiO_x_-based GIE-trapping devices can only be used for negatively charged nanoparticles. To utilize these devices for positively charged nano-objects and broaden the scope of GIE trapping, the device surface has to be modified to acquire a net positive surface charge density. We have previously reported positive single nanoparticle trapping in a surface-modified glass-based GIE-trapping device, where a conventional glass-based integrated nanofluidic device was functionalized using polyelectrolyte solution^20^. However, the surface modification of a glass-based nanofluidic device is a cumbersome and time-consuming process since the introduction and exchange of solutions rely on capillary action and diffusion of liquid molecules, respectively. Here, we report on overcoming this issue using polydimethylsiloxane (PDMS)-based integrated nanofluidic devices that allow faster multilayer functionalization and alteration of the surface electrostatic charge. Soft-elastomeric GIE-trapping devices reduce both the fabrication time and functionalization time from nearly 5 days to just a few hours, leading to a nearly one order of magnitude change in time. Surface modification of PDMS-based devices can be performed in minutes, opening up a new possibility of selective functionalization of device surfaces for charge-selective and area-specific trapping of charged nanoparticles. It can also be used to separate positively and negatively charged nanoparticles before trapping, which can work as a particle separation machine. Furthermore, PDMS-based devices enable tunable trapping and release of nanoparticles during experiments owing to the low elastic modulus of PDMS^25–27^. This allows the study of multiple batches of particles with the same GIE-trapping device, giving more statistical information during a single experiment. Other chemical and physical properties of PDMS, such as the biocompatibility, gas permeability, and optical transparency, further enable these devices to be used in drug discovery, biosensing, and disease diagnosis.

In this report, we present fabrication and multilayer surface functionalization procedures for PDMS-based GIE-trapping devices and their usage in single particle trapping for negatively charged nanoparticles. A comparison of experimental and numerically calculated particle trapping results is presented along with the impact of the size of the nanopocket in single nanoparticle GIE trapping. The novelty of this work lies in the following areas:

1. Functionalization of PDMS-based nanofluidic devices to alter the surface charge density of the device surface.
2. Simplification of the functionalized GIE-trapping devices for high-throughput particle trapping.
3. Improvement of the production of functionalized GIE-trapping devices for positively charged particle trapping.
4. Opening up of the selective surface functionalization prospects of a nanofluidic device for GIE trapping of both positively and negatively charged particles.

## Results

### Electrostatic single particle trapping

Single particle confinement experiments were conducted using PDMS-based GIE-trapping nanofluidic devices with and without surface functionalization to analyze the effect of surface modification on the contact-free confinement of a single nano-object. Surface functionalization was carried out using polyelectrolytes PEI (poly(ethyleneimine))^28–32^ and PSS (poly(sodium 4-styrenesulfonate)), as presented in section “Functionalized PDMS Device”. For experiments, a particle sample solution was prepared as described in section “Particle Sample Preparation” using negatively charged 80 nm diameter gold nanoparticles in DI water in place of buffer solution. The obtained salt concentration of the final particle solution was in the range of ∼0.02–0.05 mM. Particle samples were always prepared fresh on the day of the experiment to avoid particle agglomeration.

PDMS fluidic devices with and without surface functionalization were obtained using a PDMS mold and cover glass, as described in sections “Functionalized PDMS Device” and “Nonfunctionalized PDMS Device”, respectively. For functionalization, two layers - the first cationic layer (PEI) and second anionic polyelectrolyte layer (PSS) - were adsorbed on the PDMS mold and the cover glass surface. The two-layer (PEI and PSS) functionalization of the PDMS and glass surface resulted in a net negative surface charge density of the substrates.

**Fig. 1:**
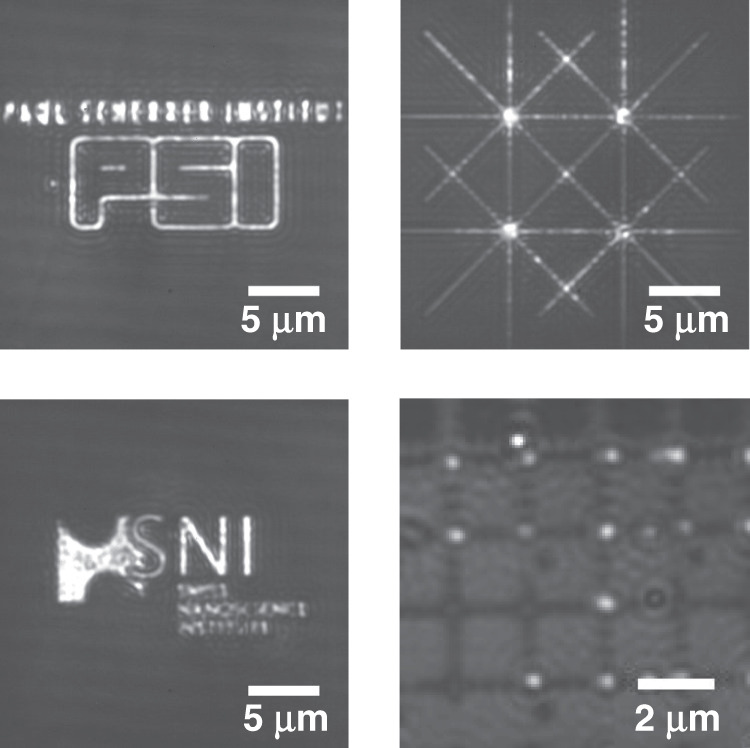
GIE trapping in nanostructures. Nanoparticle trapping in various nano- and microstructures imbedded in nanochannels. The depths of nano/microindentations are 80 nm in the case of the Unibasel, PSI and SNI logos and 100 nm in the case of the grid; the channel height is 160 nm

**Fig. 2:**
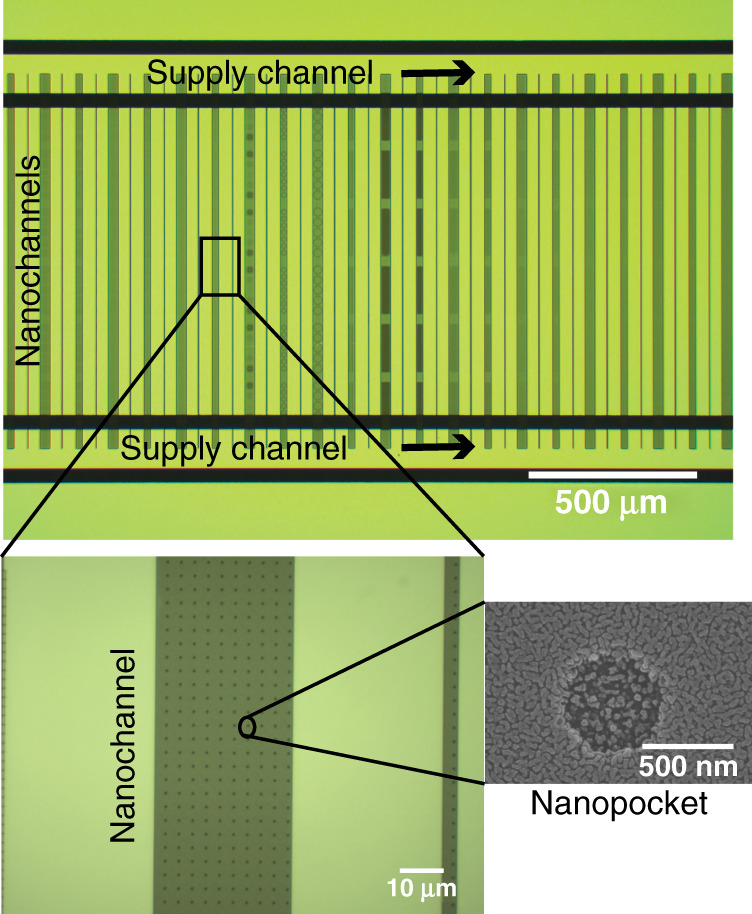
Patterning of the glass master for the GIE-trapping device development. Patterned glass substrate with supply channels connected to multiple nanochannels imbedded with nanoindentations of different geometries along with circular nanopockets with diameters of 200 nm and 500 nm

**Fig. 3:**
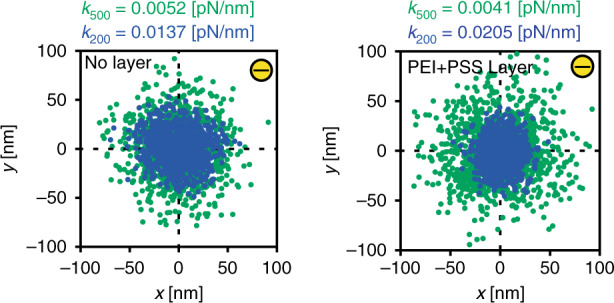
Scatter plots of the trapped nanoparticles. Scatter plots of negatively charged single 80 nm diameter gold nanoparticles confined in a contact-free manner in 200 nm (blue) and 500 nm (green) diameter pockets inside a native device (left) and the polyethyleneimine and poly(sodium 4-styrenesulfonate)-functionalized (right) PDMS device. The spread of the scatter plots of the trapped particles in the 500 nm and 200 nm pockets shows lower trapping strength of the potential trap for 500 nm nanopockets than for 200 nm pockets. The calculated stiffness constants (*k*) for the functionalized and native device were comparable for both the 200 nm and 500 nm nanopockets, denoting the homogeneous surface functionalization of the device

For particle confinement, various geometries of nanostructures can be used, as shown in Fig. 1, enabling single particle to multiparticle trapping depending on the size and geometry of the nanoindentations. These nanoindentations can be modified according to the requirements of the experiments and the types of objects that need to be trapped. In our quantitative study, experimental trapping data were collected for circular nanoindentations with diameters of (i) 200 nm and (ii) 500 nm and a depth of 100 nm, as shown in Fig. 2. To collect particle dynamics information for the trapped particles, images of the trapped nano-objects were collected using the interferometric scattering (iSCAT) detection method. Collected images were further processed to calculate the trapping stiffness of the potential trap as described in section “Particle Detection and Tracking”. Scatter plots of the trapped single nanoparticles were obtained using the *x* and *y* coordinates of the center of the confined particle, as shown in Fig. 3. Using the particle trajectory inside the potential trap, the radial mean square displacement (*MSD*_*r*_) of the particle was calculated, which reaches a plateau for a confined particle^33^. As mentioned in section “Particle Detection and Tracking”, the *MSD*_*r*_ value at the plateau is related to the radial trapping stiffness constant as $\left[ {MSD_r} \right]_{plateu} = 4k_{\mathrm{B}}T/k_r$, thus giving quantitative information about the strength of the potential trap^26,34^.

**Fig. 4:**
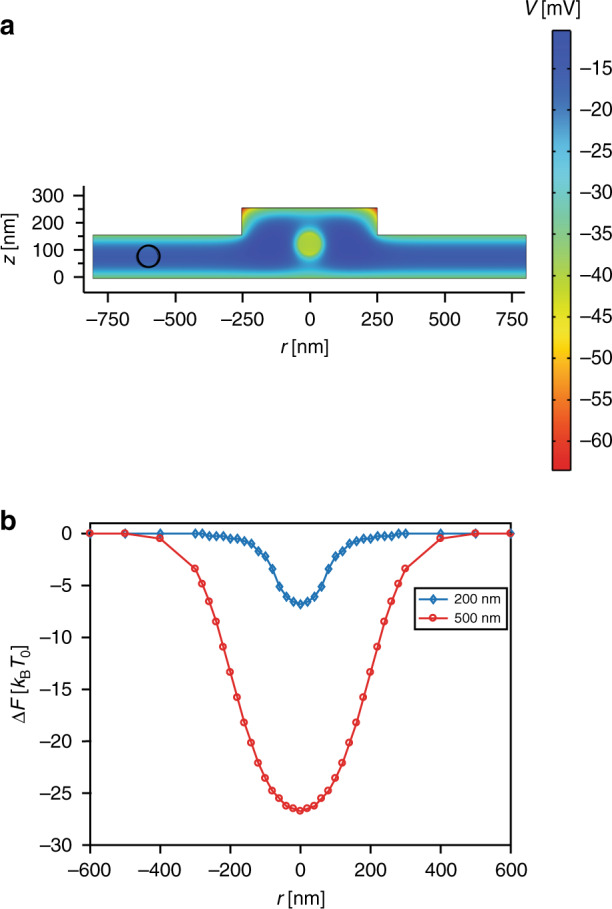
Electrostatic simulation result for the GIE trap. **a** 2D electrostatic potential distribution along the *yz-*plane at *x* = 0 for channel height = 160 nm, pocket depth = 100 nm, pocket diameter = 500 nm, salt concentration = 0.03 mM, and particle diameter = 80 nm. 3D COMSOL simulations were performed for different particle locations inside and outside of the pocket ranging from *r* = 0 nm (bright circle) to *r* = 600 nm (black circle). A potential distribution was used for the calculation of the system free energy as a function of the particle position relative to the center of the nanotrap (i.e., *r* = 0). **b** Helmholtz free energy distribution with respect to the radial position (*r*) of the trapped nanoparticles inside nanopockets with 200 nm (blue curve) and 500 nm (red curve) diameters. The difference in the free energy of the system was calculated with respect to the Helmholtz energy of the system with the particle at *r* = 600 nm

In experiments, the measured radial stiffness constants of trapped nanoparticles were comparable for native and surface-modified fluidic devices, denoting a homogeneous surface charge density of the functionalized device surface, which was equivalent to the surface charge density of the native device. Furthermore, we observed that nanoparticles had stronger trapping in 200 nm circular pockets than in 500 nm pockets, as shown in Fig. 3, where the radial stiffness constant of negatively charged 80 nm diameter particles was higher for a 200 nm pocket than for a 500 nm pocket. This is due to the broadening of the potential well in the case of the 500 nm pocket^35^. To further explain the impact of the pocket diameter, we conducted COMSOL simulations for a GIE trap with a negatively charged 80 nm diameter particle at different radial locations inside the trap and nanochannel, as shown in Fig. 4. In the simulations, the Helmholtz free energy (*F*) of the entire system^35–37^ was calculated for different radial locations of the nanoparticle using the self-energy of the system including the particle (*U*) and the entropy of the system $({\Delta} S):\,F = U - T{\Delta} S$*, w*here *T* is the system temperature in Kelvin.

The self-energy of the system was calculated using the electrostatic potential $(\psi )$ inside the GIE trap:

1 $$U = \frac{1}{2}\varepsilon _r \varepsilon _0 {\int_V} {(\nabla \psi )^2dV = \frac{1}{2}} {\int_A} {\sigma \psi _0dA + \frac{1}{2}} {\int_V} {\rho \psi dV}$$

and Δ*S* was calculated for monovalent-containing binary solutions such as NaCl using:

2 $$\Delta {S} = k_{\rm{B}} \int_{V} \left\{c_{\infty} \left[\left(1+\frac{e\psi}{k_{\rm{B}}T}\right) e^{-\frac{e\psi}{k_{\rm{B}}T}} + \left(1 - \frac{e\psi}{k_{\rm{B}}T}\right) e^{\frac{e\psi}{k_{\rm{B}}T}} -2 \right] \right\} dV$$

The potential distribution inside the GIE trap was calculated using the Poisson–Boltzmann (PB) equation:

3 $$\nabla \cdot \left( {\varepsilon E} \right) = \rho _v$$

where $E = - \nabla \psi$ and $\varepsilon$ is the permittivity of the solution at zero frequency. The total free charge density is $(\rho _v) = - 2c^\infty e\sinh \left( {\frac{{e\psi }}{{k_{\mathrm{B}}T}}} \right)$, where *k*_B_ is the Boltzmann constant. These simulations were solved using the electrostatics model in COMSOL, which was validated against previously published work^18,35^.

To compare the simulations with real experiments, simulations were performed with the nanoparticle situated at the location of minimum potential energy along the *z*-direction at different radial positions. The location of minimum potential energy along the *z*-axis for different radial locations was calculated prior to charged-particle-inclusive simulations by using COMSOL simulations performed with a point charge approximation of the charged particle^37^. For COMSOL simulations (COMSOL Multiphysics package 4.2), the Poisson–Boltzmann equation was solved in 3D space using the surface charge density of PDMS and glass −3 × 10^−3^ *e*/nm^2^, in agreement with the literature^26,38^, and the ionic strength of monovalent salt solution, 0.03 mM based on zeta potential measurements. The particle surface charge used in the simulations for the 80 nm diameter nanoparticle was −132 e based on the zeta potential measurement.

The device geometry used for simulations was similar to that of the real device with a 160 nm channel height, a 100 nm pocket depth, and pocket diameters of 200 nm and 500 nm, as shown in Fig. 4. The nanopockets were kept cylindrical with rotational symmetry along the *r* = 0 axis. To attain the final free energy distribution curve, 3D COMSOL simulations were performed for different particle locations inside and outside of the pocket ranging from *r* = 0 nm to *r* = 600 nm. In the lateral direction, the particle was located at the position of minimum potential energy at a fixed radial position. The *z*-locations for the minimum potential energy were calculated using 4 × 4 nm^2^ averaging of the potential distribution obtained from the point charge approximation COMSOL simulations. The simulated potential distribution for each particle location was further used to calculate the Helmholtz free energy (*F*) of the system under individual particle configurations by calculating the self-energy of the system and entropy of the system^18,35^. Employing the Helmholtz energy difference as a function of *r*, the trapping stiffness constant was calculated by ${\Delta} F = 1/2k_rr^2$ (ref. ^18^). The stiffness constants obtained using simulated results were 0.0043 [pN/nm] for 200 nm diameter pockets and 0.0027 [pN/nm] for 500 nm pockets, which match the stiffness constants measured during experiments for stable particle trapping.

As shown in Fig. 4, broadening of the pocket diameter from 200 nm to 500 nm makes the potential trap wider and deeper. A deeper potential well allows higher residence times for trapped particles, but contrary to this, broadening of the potential well reduces the stiffness constant of the trap. A broad potential well provides the trapped particle with more room for diffusion and thus results in a lower stiffness constant. However, when comparing increments in pocket diameter from 0 to 500 nm, the stiffness constant for an 80 nm particle initially increases until ≈ 250 nm and then decreases^37^.

### Single to multiparticle trapping

**Fig. 5:**
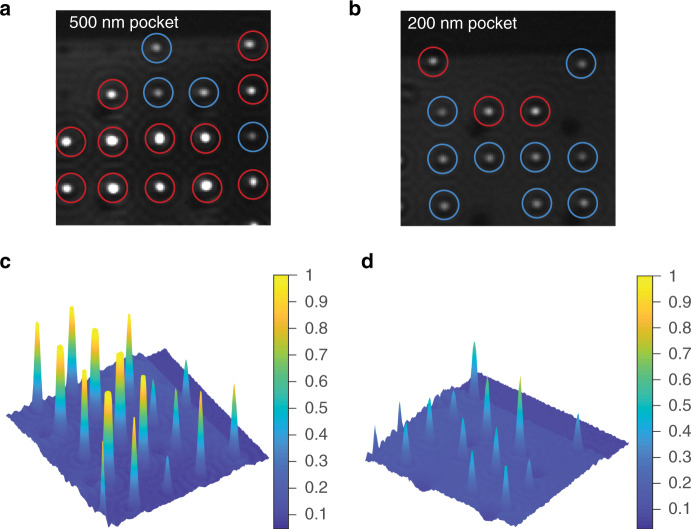
Identification of single and multiparticle occupancy in a GIE trap. Negatively charged single 80 nm diameter gold nanoparticles trapped in multiple (**a**) 500 nm and (**b**) 200 nm pockets (blue circle). Double and multioccupancy of nanoparticles was also observed (red circle) more frequently in the case of (**a**) 500 nm pockets than (**b**) 200 nm pockets. Multioccupancy was also detectable from the intensity profile of trapped nano-objects inside the nanopockets of (**c**) 500 nm and (**d**) 200 nm diameter, where the intensity was normalized with the maximum RGB value (255)

We conducted experiments for contact-free trapping of negatively charged 80 nm Au NPs using PDMS-based GIE-trapping devices. We observed that particle trapping, including the stiffness constant and residence time, is strongly influenced by the binding of PDMS with the glass surface. In the case of perfect PDMS-glass binding, we observed stable single particle trapping. In electrostatic trapping, the number of particles confined inside a nanopocket depends on the hydrodynamic radius of the particle and the diameter and depth of the nanopocket. For 80 nm diameter nanoparticles, along with single particle trapping, we observed frequent multiparticle trapping for 500 nm pockets and relatively low double or multioccupancy in 200 nm pockets, as shown in Fig. 5. Single and multiparticle occupancy in the nanopockets can be identified based on the intensity profile of collected images, as demonstrated in Fig. 5. Since the images were collected using the iSCAT method, larger particles scatter more light and thus appear brighter in the collected image. The scattering intensity is proportional to the size of the particle; thus, a multiparticle assembly gives a higher intensity than a single nanoparticle. To achieve single particle trapping throughout the GIE-trapping PDMS-based device, it is important to optimize the pocket diameter according to the hydrodynamic radius of the particle of interest inside the buffer solution that will be used for the particle trapping experiment.

### Experimental comparison for functionalized and nonfunctionalized devices

The final particle trapping depends on the surface charge density of the device surface along with the geometrical parameters of the device; therefore, the trapping stiffness constant for trapped particles in a functionalized PDMS-based device indirectly provides information on how homogeneous and quantitatively similar the surface charge density is for a surface-modified device with respect to the native device. For successful contact-free nanoparticle trapping, the device surface must be homogeneously functionalized. In the presence of an inhomogeneous functionalized surface, charged particles become stuck to the surface and do not show electrostatic trapping.

Experimental data for conventional and two-layer polyelectrolyte (PEI and PSS)-functionalized PDMS devices were compared based on the observed trapping stiffness constants of confined single nanoparticles. The stiffness constant of a trapped object denotes the strength of confinement and the scope to which a particle moves inside the electrostatic trap.

**Fig. 6:**
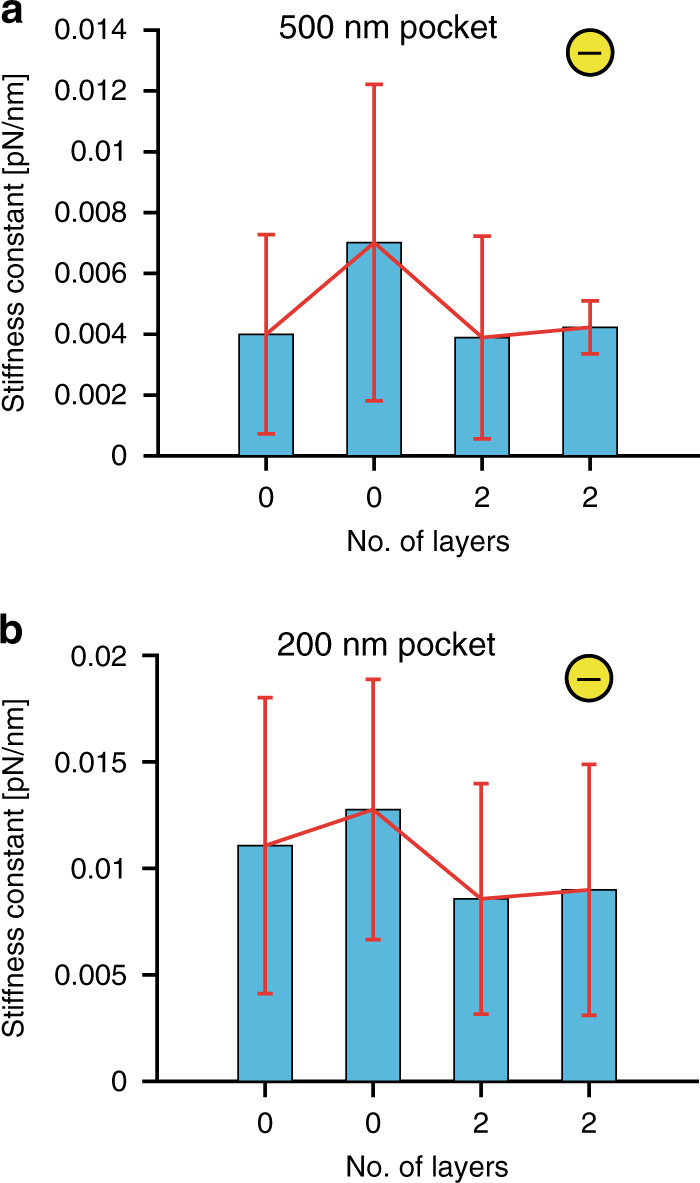
Trapping stiffness constant distribution of the trapped charged single nanoparticles. Comparison of the trapping stiffness constant obtained for negatively charged single 80 nm gold nanoparticles trapped in (**a**) 500 nm and (**b**) 200 nm pockets for both two-layer polyelectrolyte functionalized and nonfunctionalized (0-layer polyelectrolyte) PDMS devices with a 160 nm channel depth and a 100 nm pocket depth. Two datasets were collected from two different experiments to show the distribution variation of the single nanoparticle trapping stiffness constant between different experiments

We compared experimental data for 500 nm and 200 nm diameter nanopockets. As shown in Fig. 6, a single trapped particle in 500 nm pockets had a larger spread of the stiffness constant due to higher freedom of movement inside the potential trap, which can be understood from the simulation results shown in section “Electrostatic Single Particle Trapping”. Experimentally, we observed that for 500 nm pockets, loosely trapped 80 nm single particles moved within a 200 nm distance from the pocket center and had a stiffness constant in the range of $\sim\!0.0004\! - \!0.0006$ [pN/nm], whereas particles trapped stably inside the pocket diffused in the range of 150 nm, 130 nm, and 100 nm from the center of the pocket and had *k*_*r*_ in the range of $\sim\!0.0008\!-\!0.002$ [pN/nm], $\sim\! 0.002\!-\!0.003$ [pN/nm], and $\sim\! 0.002\! -\! 0.004$ [pN/nm], respectively. For strongly trapped nanoparticles with diffusion in the range of ≤75 nm around the pocket center, *k*_*r*_ was $\sim\! 0.005\! -\! 0.022$ [pN/nm]. Similarly, for 200 nm pockets, 80 nm gold nanoparticles loosely trapped in 200 nm pockets were confined in the range of 100 nm and showed a stiffness constant in the range of $\sim\! 0.003\! -\! 0.006$ [pN/nm]. For stable trapping, particles diffused over distances of ≤70 nm and ≥50 nm around the pocket center, with $k_{\mathrm{r}}\sim 0.004\! -\! 0.01$ [pN/nm], and for strong confinement, particle diffusion was in the range of <70 nm and ≥30 nm with $k_{\mathrm{r}}\sim 0.01\! -\! 0.03$ [pN/nm]. This observation was valid for both two-layer functionalized and nonfunctionalized PDMS devices.

During particle trapping, the particle confinement inside the potential well varies depending on the hydrodynamic diameter and net surface charge of the particle^37^ along with the slight variations (in the nanometer range) in the geometry of the potential trap, leading to a distribution of the trapping stiffness constant, as shown in Fig. 6. The nanoparticles used for experiments had a coefficient of variation (CV) for the diameter of nearly 8% and showed a distribution of the net particle surface charge during zeta potential measurements (CV ∼ 11%), leading to a distribution of the experimental data.

When geometrical optimization is required to attain stronger particle trapping, it is important to understand the possible causes of the distribution of the stiffness constant, which are mainly related to physical phenomena and experimental conditions. When a particle reaches the inside of a potential trap, it goes through a momentum relaxation process related to its ballistic motion. This momentum relaxation time (*r*_*p*_) related to ballistic motion can be in the range of a few picoseconds to a few nanoseconds. In our case, *r*_*p*_ is ∼8 ns. The ballistic motion of the charged particle occurs primarily before the particle is trapped inside the potential well. Once the charged particle is trapped, it undergoes an oscillatory motion in the harmonic well of the potential trap, which is mainly governed by diffusion instead of the particle inertia^39^. The relaxation time (*τ*_R_) of the charged particle inside the potential well is related to the viscous drag from the fluid in the device. In our case, the relaxation time (*τ*_R_) of an 80 nm diameter AuNP inside a 200 nm diameter pocket is ∼0.15 ms and inside a 500 nm diameter pocket is ∼0.25 ms. The sampling rate (∼9 ms) and integration time (1 ms) of the detection camera in our experiments are much slower than the above two relaxation times. This implies that the experimental measurements are not able to resolve the momentum relaxation dynamics of the particle and that particle motion is captured under equilibrium conditions. Since the exposure time (1 ms) is larger than the momentum relaxation time of the particle inside the potential trap, the spatial averaging of the particle motion over the larger integration time causes motion blur, leading to a larger measured stiffness constant than the actual stiffness constant of the particle^40–42^. Therefore, the particle trapping looks relatively stiff compared to its true trapping stiffness. The combination of all these factors leads to a distribution of the measured stiffness constant.

The range of obtained stiffness constants for single particle trapping in both 500 nm and 200 nm pockets for functionalized and nonfunctionalized PDMS devices, as shown in Fig. 6, demonstrates comparable particle trapping inside the PDMS device before and after functionalization. It was noted that in the case of unsuccessful two-layer functionalization, charged particles became stuck on the glass and PDMS surfaces due to electrostatic attraction forces. Only in the case of homogeneous surface functionalization was the performance of the devices maintained.

## Discussions

The presented experimental and simulation results confirm that geometry-induced electrostatic trapping for nanoparticles depends on the trap dimensions such as the trap diameter for a constant channel height and a fixed salt concentration of the solution used. It has furthermore been demonstrated that confinement of nano-objects inside nanoindentations can be measured in terms of the radial stiffness constant (*k*_r_), which can be used to identify how strongly a particle is trapped inside the electrostatic potential trap. However, particle trapping can vary based on the size and net surface charge of the particle and the depth and width of the electrostatic trap inside the nanopockets. Therefore, it is important to optimize the device geometry based on the experimental requirements to attain stable high-throughput particle trapping.

In this work, we demonstrated successful electrostatic contact-free trapping for charged single nanoparticles in a multilayer polyelectrolyte-functionalized PDMS-based nanofluidic device. The two-layer functionalized devices demonstrated trapping efficiency comparable to that of the nonfunctionalized device and numerical calculations, indicating that the overall achieved surface charge density is quantitively similar to that of the native PDMS surface and homogeneous enough to allow contact-free trapping of 80 nm diameter particles. This further demonstrated that the functionalization procedure can be used for single-layer polyelectrolyte functionalization to achieve a positively charged device surface for trapping of positively charged nanoparticles without affecting the device functionality. This broadens the scope of the electrostatic trapping method to positively charged nano-objects using functionalized PDMS-based nanofluidic devices.

The presented surface modification method for PDMS-based nanofluidic devices has not only improved the surface modification process for nanofluidic devices but also reduced the surface functionalization time by nearly 10 times. The use of PDMS-based GIE-trapping nanofluidic devices in combination with the presented surface-modification method has allowed the large-scale production of surface-modified nanofluidic devices. This furthermore has opened up new possibilities to trap both positively and negatively charged particles in the same device at different trapping locations by selectively functionalizing the trapping areas for net positive and negative surface charge density, respectively. This would be of immense use in the fields of medicine, disease diagnosis, biological studies, pathogen detection and water quality checks.

## Materials and methods

### Device patterning and replica molding

**Fig. 7:**
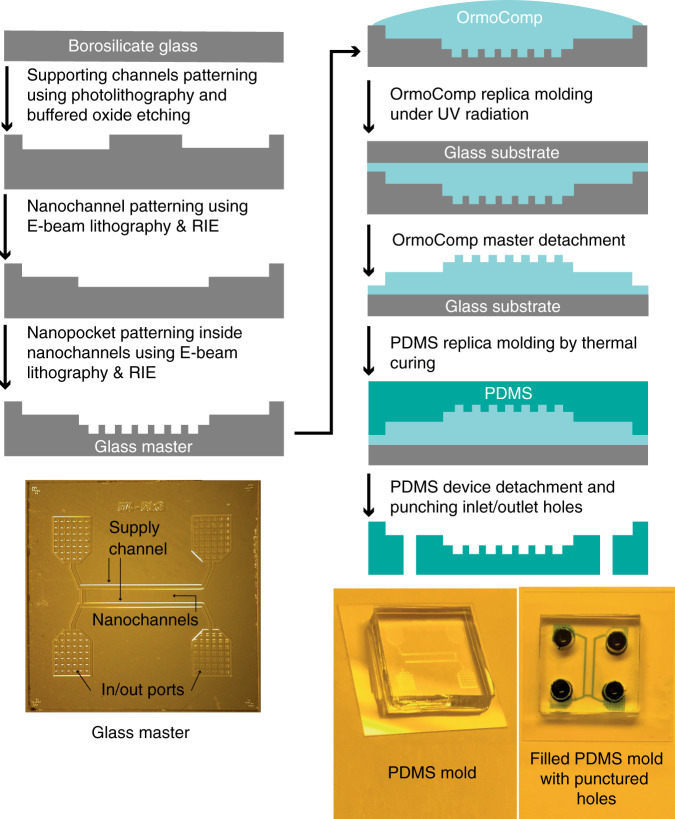
Fabrication of the PDMS-based GIE-trapping device. Schematics of the glass-master fabrication process in the left column and PDMS replica molding from the glass master using UV-curable resist OrmoComp® in the right column. Supply channels and in/out ports for the glass master are indicated with arrows. To inject a particle solution into the supply channels and nanochannels, 3 mm wide holes are punched at the location of supply ports

The devices reported here are PDMS-based GIE-trapping devices that were manufactured using replica molding^43–45^. For PDMS replica molding, a glass-based master was patterned using a combination of optical lithography, electron beam (e-beam) lithography, reactive ion etching (RIE), buffered oxide etching (BOE), and wet etching, as illustrated in Fig. 7. The detailed fabrication process for the patterned glass surface was described in our previous work^20^. The patterned glass master had dimensions of 15 × 15 mm^2^ and contained two 200 μm wide and 11 μm deep buffer supply channels with in/out ports at both ends. Both supply channels were connected by multiple 1 mm long nanochannels imbedded with cylindrical nanopockets, as shown in Fig. 2. The nanochannels were ~160 nm deep and 30 μm wide with imbedded nanopockets of diameters of 200 nm and 500 nm and a pocket depth of 100 nm.

The patterned glass master was used for replica molding of a negative polymer master, as demonstrated in Fig. 7. Before using the glass master for replica molding of a negative master, gas phase silane deposition was performed on the glass master in a vacuum chamber using a mixture of 1: 1 trichloro(1H,1H,2H,2H-perfluorooctyl)silane and (tridecafluoro-1,1,2,2-tetrahydrooctyl)dimethylchlorosilane. Silanization reduces the surface energy of the glass master, which allows easy removal of the glass master and negative polymer master after replica molding. For negative replica molding of the glass master, a 20 × 20 mm^2^ cover glass (700 μm thick BOROFLOAT^®^33 glass, SCHOTT Advanced Optics) was cleaned by sonication in acetone and isopropyl alcohol (IPA) for 10 min each, dried under a N_2_ stream, and oxygen plasma cleaned for 2 min (power = 150 W, pressure = 150 mTorr, O_2_ flow = 20 sccm, Oxford Instruments - Plasmalab80Plus). After cleaning, the cover glass was spin-coated with OrmoPrime08 (45 s, 4000 rpm (rotation per minute), 3000 rpm/s, Micro resist technology GmbH) and baked for 5 min at 180 °C.

To obtain a negative polymer master, a UV-curable resin, OrmoComp^®^ (Micro resist technology GmbH), was drop-cast on the glass master, and immediately after that, an OrmoPrime08-coated cover glass was gently placed on top of the drop; the drop was left to spread and fill inside the patterned micro- and nanostructures of the glass master. Once OrmoComp^®^ filled the inside of the patterned structures, the assembly was placed in a UV chamber (ELC-500, light exposure system, Electro Life Corporation) for UV exposure (30 mW/cm^2^ for ∼10 min). After UV curing, the glass master was removed gently, leaving cured resist stuck to the primer-coated cover glass. The negative OrmoComp^®^ master was further silanized using the same procedure as for the glass master and further used for PDMS replica molding.

To mold nanostructures of the master into PDMS, a high elastic modulus (∼410 MPa) polymer is typically required^43^; thus, a 5: 1 mass ratio mixture of the base polymer (SYLGARD^®^ 184, silicon elastomer base, Dow Corning) and curing agent (SYLGARD^®^ 184, silicon elastomer curing agent, Dow Corning) was prepared to achieve an elastic modulus of ∼3.6 MPa for cross-linked PDMS^25,46^, degassed and poured on the OrmoComp^®^ master. PDMS was further cured at 150 °C for 2 h in a digital oven (Salvis Lab) in a uniform and controlled thermal environment. After curing of the PDMS mold, the oven was switched off, and samples were allowed to cool down first inside the oven for 40 min and later outside the oven at room temperature. After cooling, PDMS molds were gently separated from the OrmoComp^®^ master. Inlet/outlet holes (3 mm in diameter) were punched in the PDMS mold, as shown in Fig. 7.

### Particle sample preparation

Experiments for single particle electrostatic trapping were conducted using negatively charged gold nanoparticles (Au NPs) with a diameter of 80 nm (BBI Solutions, EM.GC80). The purchased gold nanoparticles had intrinsic –COOH functional groups present on the particle surface. These –COOH groups are exposed to the environment and dissociate into H^+^ and COO^−^ in the aqueous solution, providing a net negative surface charge density to the nanoparticles.

Sample solutions for experiments were prepared using 1 ml of negatively charged 80 nm Au NP solution in a 1.5 ml Eppendorf tube (RNase-free Microfuge tubes, Thermo Fisher Scientific). Nanoparticles were washed by exchanging particle buffer solution with DI water in the following steps: (i) centrifuge the solution at a 2000 relative centrifugal force (rcf) for 20 mins; (ii) discard the supernatant, and resuspend the pellet in 1 ml of DI water; and (iii) repeat the previous cleaning steps (i–ii) two times, and in the last step, resuspend the pellet in 50 μl of DI water instead of 1 ml. The prepared solution had a $\sim\! 0.02\!-\!0.05$ mM salt concentration remaining from the particle buffer solution and ∼10^12^ particles/ml.

### Nonfunctionalized PDMS device

**Fig. 8:**
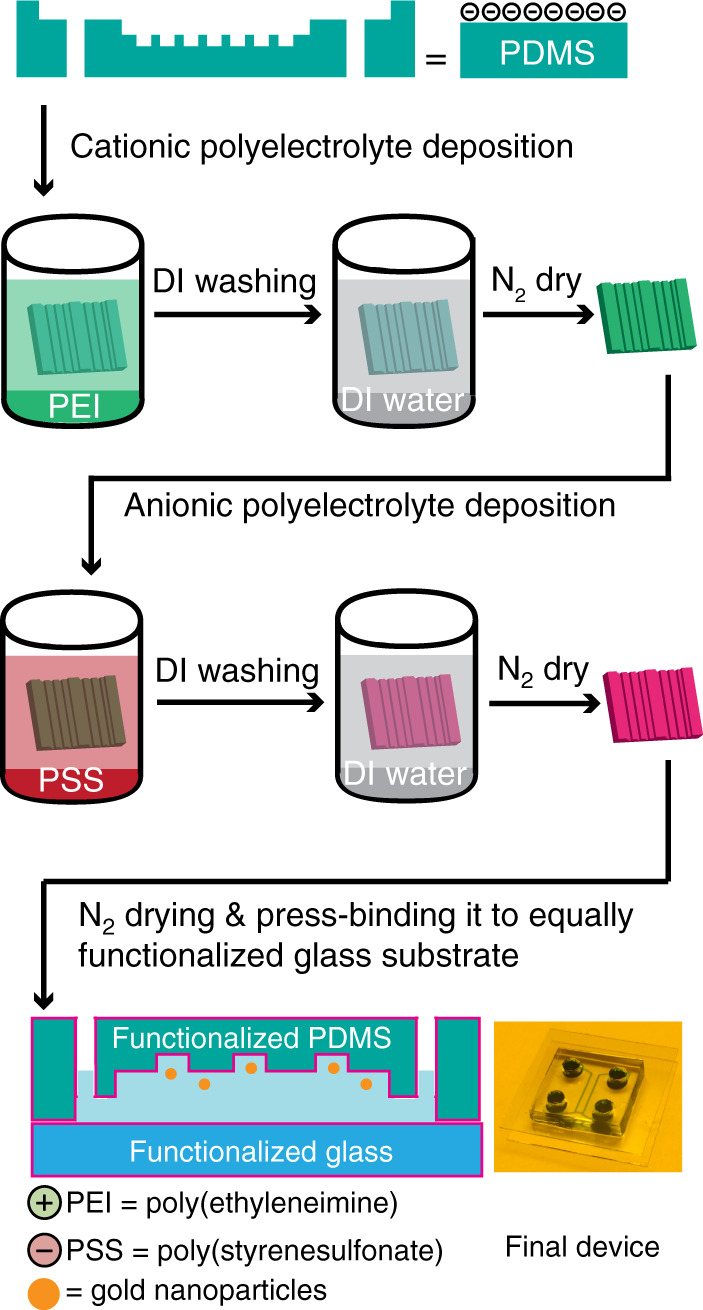
Illustration of the surface modification process. Schematics of the surface functionalization process for the polydimethylsiloxane-based nanofluidic device using cationic and anionic polyelectrolytes

The PDMS-based GIE-trapping device was made of an assembly of a patterned PDMS mold and two cover glasses (ROTH KARLSRUHE, 200 Deckgläser, 20 × 20 mm, #1), as shown in Fig. 8. Prior to device assembly and functionalization, the cover glasses were cleaned by sonication in acetone (15 min), IPA (15 min), and DI water (15 min) sequentially. Immediately after this step, the cover glasses were immersed for 20 min in a freshly prepared piranha solution, which was a mixture of 2: 1 sulfuric acid (H_2_SO_4_) and 30% hydrogen peroxide (H_2_O_2_). Then, the cover glasses were thoroughly washed under a DI water jet and later dried under a nitrogen (N_2_) gas stream.

In the case of native (nonfunctionalized) PDMS devices, both the PDMS mold and the cleaned cover glasses were air-plasma activated (∼42 s, 80% power - full power 100 W, 1.0 mbar, Diener electronic, Femto). Within 2 min, nanochannels of the patterned PDMS mold were filled with 0.25 μl of particle solution and placed on a cover glass, keeping the patterned side down. To achieve strong covalent PDMS-glass binding, the PDMS mold was pressed gently. Immediately after that, all inlet/outlets were filled with the buffer solution to avoid drying of the nanochannels. After inlet/outlet filling, the other cleaned air-plasma activated cover glass was placed on top of the PDMS mold to avoid evaporation of solution from in/outlet ports, as shown in Fig. 8. The obtained device with filled particle solution was directly used for the experiment ∼45 min after preparation to allow the solution to reach an equilibrium state.

### Functionalized PDMS device

A PDMS mold acquires a negative surface charge density in the presence of an aqueous solution due to the self-dissociation of terminal silanol groups^38,47^. The acquired negative surface charge density permits contact-free electrostatic trapping only for negatively charged nano-objects. To use the same device for positive-particle confinement, the net surface electric charge needs to be changed to positive by surface functionalization^20^. For a homogeneous surface charge density, multilayers of alternating cationic and anionic polyelectrolytes can be used for surface functionalization^48^. To compare the GIE-trapping efficiency of the functionalized device with that of the original device, we performed surface functionalization to change the surface charge first from negative to positive and then from positive to negative. Thus, we used two layers of polyelectrolytes, poly(ethyleneimine) (PEI) and poly(styrenesulfonate) (PSS), to attain a homogeneous and long-lasting negative surface charge density inside the PDMS GIE-trapping device, as shown in Fig. 8. The combination of PEI, a weak positive polyelectrolyte, and PSS, a strong negative polyelectrolyte, has been proven to form a stable polyelectrolyte bilayer with a self-healing property^49^.

To achieve uniform surface functionalization and homogeneous surface charge density inside the PDMS-based GIE-trapping device, one PDMS mold and two cover glasses were identically functionalized prior to device assembly. Since both PDMS and glass attain negative surface charge densities in an aqueous environment, the first layer of polyelectrolyte used was a cationic layer. To assist polycation adsorption on PDMS and the glass surface, both the PDMS mold and cover glasses were air plasma activated (42 s, 80% power, 1.0 mbar). Immediately after that, the PDMS mold and glasses were immersed in a 4 mg/ml solution of a cationic polyelectrolyte for 9 min. For the cationic polyelectrolyte, branched poly(ethyleneimine) (PEI, 50% w/v in H_2_O, Fluka Analytical, Sigma-Aldrich) solution was used due to its high cationic charge density. The branched PEI polymer consists of primary, secondary and tertiary amine groups. In general, every third group in PEI is an amine with high affinity towards protonation^50,51^. After 9 min of incubation in PEI solution, both the PDMS mold and the cover glasses were washed thoroughly with Milli-Q water and dried under a N_2_ stream. Adsorption of PEI polyelectrolyte on the PDMS and glass surfaces was verified through particle trapping experiments using positively charged nanoparticles. Immediately after drying, both the PDMS mold and the cover glasses were immersed in a 4 mg/ml anionic polyelectrolyte solution of poly(sodium 4-styrenesulfonate) (PSS, 30% w/v in H_2_O, M_w_ ∼ 70,000 Da, Sigma-Aldrich) for 9 min. After polyanion adsorption, both the PDMS and the cover glasses were washed thoroughly with Milli-Q water and N_2_ dried. Immediately after drying, nanochannels on the functionalized PDMS mold were filled with nanoparticle solution, and the filled PDMS mold was placed on a functionalized glass and pressed gently to achieve PDMS mold and glass binding. To avoid drying of nanochannels, all inlets and outlets were filled with buffer solution, and the other identically functionalized cover glass was placed on top of the PDMS mold to obtain the final device for the experiment, as shown in Fig. 8. Here, we used deionized (DI) water (18 MΩ/cm^−1^) in place of buffer solution for particle trapping experiments.

In the functionalization procedure, cleaning after polyelectrolyte adsorption played a crucial role in multilayer functionalization of PDMS and glass surfaces. Thus, thorough washing was required to wash away nonadsorbed polyelectrolytes and for proper physisorption of the consecutive polyelectrolyte layer. Using the adsorption of polycations and polyanions alternatively, one can achieve multiple layers of polyelectrolytes^48^.

### Particle detection and tracking

**Fig. 9:**
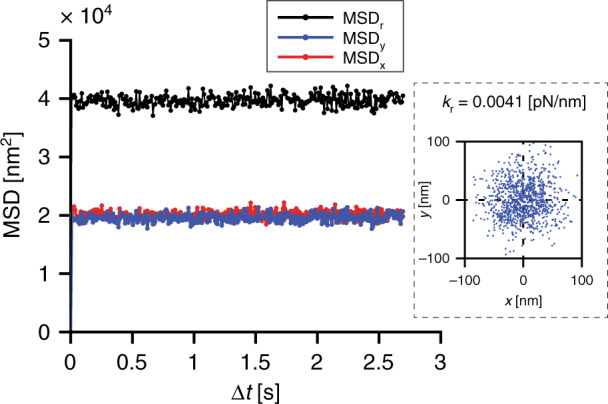
Mean square displacement of a trapped particle. Mean square displacement plot as a function of lag time (Δ*t*) of a negatively charged 80 nm diameter particle trapped inside a 500 nm wide nanopocket in a functionalized PDMS device. The *x* and *y* coordinates of the particle center inside the potential trap (inset) are used to calculate the *MSD*_x_ (red), *MSD*_y_ (blue) and *MSD*_r_ values. The *MSD*_r_ value at the plateau is then used to calculate the stiffness constant of the trapped particle

Trapped nanoparticles inside a GIE-trapping nanofluidic device were imaged using a home-built iSCAT microscope^52–54^. The detection relies on the interference of the light scattered from the trapped nano-object and light reflected from the interface of the buffer solution and the substrate. The iSCAT setup was used for particle detection and particle motion recording as described in previous works^17,20,53^. The images were recorded with an exposure time of 1 ms and an acquisition rate of 111 Hz using a 300 mW, 532 nm laser-pumped solid-state laser (MGL-III-532, CNIlaser). Recorded images were used to determine the particle center and its displacement in each frame through Gaussian fitting of the particle intensity profile^39,53^. The obtained two-dimensional *x* and *y* coordinates for the particle center were further used to calculate the radial mean square displacement (*MSD*_*r*_) of the trapped nano-object inside a nanopocket and eventually obtain the stiffness constant^39^, as shown in Fig. 9. *MSD*_r_ = $\langle\left[ {{\Delta} r\left( {{\Delta} t} \right)^2} \right]\rangle$, where $r = \sqrt {x^2 + y^2}$, was calculated as a function of lag time ${\Delta} t$. The *MSD*_*r*_ value reaches a plateau at large lag times for a trapped particle due to its restricted motion inside a nanopocket. The *MSD*_*r*_ value at the plateau, $\left[ {MSD_r} \right]_{plateu}\, = \langle\left[ {{\Delta} r} \right]_P^2\rangle$, is directly related to the radial stiffness constant $\left( {k_{\mathrm{r}}} \right)$ of the electrostatic potential trap inside the nanopocket by $\left[ {MSD_r} \right]_{plateu} = 4k_{\mathrm{B}}T/k_{\mathrm{r}}$, where $k_{\mathrm{B}}$ is the Boltzmann constant and *T* is the absolute room temperature^34^.

To better understand the electrostatic potential trap, the mean residence time of the trapped particle inside the trap (Kramers time = $\bar \tau _{\mathrm{k}}$) and potential depth (*Q*) of the trap are also required in addition to the stiffness constant. The Kramers time and potential depth of the trap are related as $\bar \tau _{\mathrm{k}} \cong \tau _{\mathrm{R}}e^{Q/k_{\mathrm{B}}T}$, where $\tau _{\mathrm{R}}$ is the relaxation time in the potential well, which is equivalent to the time an untrapped particle takes to freely diffuse through a distance corresponding to the width of the potential well^26^. Relaxation time values can be calculated using $\tau _{\mathrm{R}} = k_{\mathrm{B}}T/Dk_r$, where the diffusion constant for a particle with diameter *d* is $D = k_{\mathrm{B}}T/3\pi \eta d$, and $\eta$ is the dynamic viscosity of the solution^26,34,55^. When particle trapping measurements are performed under equilibrium conditions with exposure times larger than $\tau _{\mathrm{R}}$, the MSD is a flat plateau with the $MSD_r({\Delta} t)$ value reaching $\left[ {MSD_r} \right]_{plateu}$, as presented in Fig. 9. However, for exposure times shorter than $\tau _{\mathrm{R}}$, the $MSD_r({\Delta} t)$ value monotonically increases and reaches a plateau.
